# A Systematic Review of the Effect of Oral Rinsing with H_2_O_2_ on Clinical and Microbiological Parameters Related to Plaque, Gingivitis, and Microbes

**DOI:** 10.1155/2020/8841722

**Published:** 2020-10-31

**Authors:** Francisco Wilker Mustafa Gomes Muniz, Juliano Cavagni, Gerson Pedro José Langa, Bernal Stewart, Zilson Malheiros, Cassiano Kuchenbecker Rösing

**Affiliations:** ^1^Department of Periodontology, Federal University of Pelotas, Pelotas, Brazil; ^2^Department of Periodontology, Federal University of Rio Grande do Sul, Porto Alegre, Brazil; ^3^Colgate-Palmolive Technology Center, Piscataway, NJ, USA

## Abstract

**Background:**

Hydrogen peroxide (H_2_O_2_) has been used for more than a century clinically to control plaque and gingival inflammation, with unclear supporting evidence.

**Aim:**

The aim of the present systematic review of the literature is to assess the effect of mouth rinses with H_2_O_2_ on dental plaque, gingival inflammation, and oral microorganisms.

**Methods:**

Five databases (PubMed, Scopus, Embase, Cochrane Library, and Web of Science) were searched with the following focused question: what is the effect of hydrogen peroxide, in comparison to chlorhexidine or to a placebo solution, in oral microbiota control, dental plaque, and gingival inflammatory outcomes? Two independent examiners retrieved the articles and evaluated the evidence.

**Results:**

The majority of included studies were performed with 1.5% H_2_O_2_. Results related to plaque accumulation generally demonstrate a slightly better effect of H_2_O_2_ as compared to placebo mouth rinses, however with a lower performance as compared to chlorhexidine. In terms of gingival inflammation, H_2_O_2_ performs better than placebo and more clearly demonstrates an anti-inflammation effect. No studies evaluated the effect of H_2_O_2_ against viruses or fungi. In terms of bacteria, H_2_O_2_ demonstrates an antibacterial effect.

**Conclusion:**

Rinsing with H_2_O_2_ has the potential to affect plaque, gingivitis, and oral bacteria, as compared to placebo. However, the antibacterial results are not comparable to the performance of chlorhexidine.

## 1. Introduction

Hydrogen peroxide (H_2_O_2_) mouthwashes have been used for a long time [1]. They have been utilized in an attempt to complement mechanical plaque control methods as well as to prevent/control oral infections [2]. However, the evidence supporting its use is not unequivocal even though it is still used by a number of professionals.

In 2011, Hossainian et al. [3] published a systematic review to evaluate the effect of H_2_O_2_ mouthwashes on the prevention of plaque and gingival inflammation. The focused question of such review was as follows: “what are the effects of oxygenating mouthwashes on plaque accumulation and gingival inflammation parameters in adults, when compared with positive or negative controls mouthwashes or no oral hygiene, when used as a monotherapy or as an adjunct in daily oral hygiene?” Surprisingly, the number of included studies was relatively low (*n* = 12, in which only 5 were specifically formulated with H_2_O_2_). The other 7 studies were related to other oxygenating agents. The results of the review demonstrated that mouthwashes containing H_2_O_2_ do not consistently prevent plaque accumulation when used as a short-term monotherapy.

Recently, the effect of H_2_O_2_ on viruses has become a renewed interest due to the COVID-19 pandemic. H_2_O_2_ mouth rinses are being recommended as a preprocedural rinse, as well as a regular rinsing solution with the aim of diminishing contamination possibilities by the new coronavirus. A number of associations, including the American Dental Association, are recommending the use of H_2_O_2_ mouth rinses as prerinses prior to procedures [4–6]. However, the evidence for the use of H_2_O_2_ for oral antiviral purposes is virtually nonexistent. Most of the evidence only demonstrates the potential of H_2_O_2_ to disinfect surfaces [7].

New studies have been conducted and published after the systematic review of Hossainian et al. [3]. Hence, the existing review [3] could be broadened to consider these additional studies, especially in this particular moment. Therefore, the aim of this study is to systematically review the literature, assessing the effects of H_2_O_2_ mouth rinses in controlling dental plaque, gingival inflammation, and oral microbiota.

## 2. Materials and Methods

The focused question of the present study was as follows: “what is the effect of hydrogen peroxide, in comparison to chlorhexidine or to a placebo solution, in oral microbiota control, dental plaque, and gingival inflammatory outcomes?”

In order to be included, the study must fulfill all of the following inclusion criteria:

- Clinical trials with humans of any age.
- Test group: individuals that used, at least one time per day, hydrogen peroxide mouthwash. Any concentration of hydrogen peroxide was accepted.
- Control group: individuals that used, at least one time per day, a placebo or chlorhexidine mouthwash. Any concentration of chlorhexidine was accepted.
- Outcomes: any oral microbiological, plaque index, or gingival index analysis.

No restriction to language or date of publication was imposed. Studies that used both chlorhexidine and hydrogen peroxide in the same group were excluded. Studies that involved outcomes assessed in dental implants were also excluded.

A search strategy was performed, up to April 23, 2020, in five databases: PubMed, Scopus, Embase, Web of Science, and Cochrane Central Register of Controlled Trials (CENTRAL). The search strategy performed in PubMed database is expressed as follows:

  Terms for hydrogen peroxide: Hydrogen Peroxide [MeSH terms] OR Hydrogen Peroxide [text word] OR Peroxides [text word] OR H_2_O_2_ [text word] OR Hydroperoxide [text word] OR Superoxol [text word] OR Oxydol [text word] OR Perhydrol [text word] OR oxidizing agent [text word] OR Bocasan [text word] OR Amosan [text word] OR Peroxyl [text word] OR Ascoxal [text word] OR peroxyborate [title/abstract]
  Terms for mouthwashes: Mouthwashes [MeSH terms] OR mouthwashes [text word] OR mouthrinses [text word] OR mouthwash [text word] OR mouthrinse [text word] OR rinse [text word] OR Mouthwashes [pharmacological action] OR mouth rinse [text word] OR mouth rinses [text word] OR Mouth Bath [text word] OR Mouth Baths [text word] OR Mouth Wash [text word] OR Mouth Washes [text word] OR Oral Sprays [MeSH terms] OR Oral Sprays [text word] OR spray [text word]
  Terms to assess all outcomes: microbiology [MeSH terms] OR microbiology [text word] OR Bacteria [MeSH terms] OR Bacteria [text word] OR Streptococcus mutans [text word] OR Porphyromonas gingivalis [text word] OR Aggregatibacter actinomycetemcomitans [text word] OR Tannerella forsythia [text word] OR Fusobacterium nucleatum [text word] OR Treponema denticola [text word] OR streptococcus sobrinus [text word] OR Streptococcus sanguis [text word] OR bacterial strain [text word] OR fungi [text word] OR fungi [MeSH terms] OR fungus [text word] OR Mold [text word] OR Candida [text word] OR Oral pathogens [text word] OR oral microorganisms [text word] OR antimicrobial [text word] OR antibacterial [text word] OR minimal inhibitory concentration [text word] OR MIC [text word] OR ATCC [text word] OR Type Culture Collection [text word] OR Saliva [MeSH terms] OR saliva [text word] OR dental caries [MeSH terms] OR Dental Caries [text word] OR dental plaque [MeSH terms] OR Dental plaque [text word] OR plaque [text word] OR biofilms [MeSH terms] OR biofilm [text word] OR dental deposit [text word] OR antiplaque [text word] OR Dental Plaque Index [MeSH terms] OR dental plaque index [text word] OR Oral Hygiene [MeSH terms] OR Oral hygiene [text word] OR Quigley-Hein Index [text word] OR Silness Loe index [text word] OR Oral Hygiene Index [MeSH terms] OR Oral hygiene index [text word] OR antigingivitis [text word] OR Gingivitis [MeSH terms] OR gingivitis [text word] OR gingival inflammation [text word] OR Bleed∗ [text word] OR gingival index [text word] OR gingival bleeding [text word] OR bleeding on probing [text word] OR papillary bleeding [text word] OR Periodontal Index [MeSH terms] OR Periodontal index [text word] OR periodontal diseases [MeSH terms] OR periodontal diseases [text word] OR periodontitis [text word]
  Terms for hydrogen peroxide AND terms for mouthwashes AND terms to assess all outcomes

An adaptation of the abovementioned search strategy was performed in the other databases. Two researchers independently selected the studies and extracted the data in a spreadsheet specifically developed for this study (CKR and FWMGM). Regarding study selection, both screening (title and abstract analyses) and eligibility (full-text analysis) phases were performed independently. All discrepancies were solved between the researchers by discussion.

In addition, hand search was performed in the list of references of the included studies and in previously published literature reviews [3]. Studies from the last 30 years were hand searched in the following journals: Journal of Clinical Periodontology, Journal of Periodontology, Journal of Periodontal Research, and Journal of Dental Research. Searches for grey literature were also performed in the Clinical Trials (clinicaltrials.gov) and Google Scholar databases, using an adaptation of the abovementioned search strategy. All the corresponding authors of the included studies were contacted by e-mail in order to detect other potentially relevant clinical trials. In addition, manufactures were contacted to supply information about other published, unpublished, or ongoing research studies using H_2_O_2_.

The risk of bias of all randomized clinical trials was assessed by the RoB2 tool, as recommended by Cochrane [8]. Randomization process, deviations from the intended interventions, missing outcome data, measurement of the outcome, selection of the reported results, and overall risk of bias were assessed by two reviewers (FWMGM and CKR). In case of disagreements, a consensus was made between reviewers. A positive sign was given for an item when sufficient information was available, indicating low risk of bias, and a negative mark was used, for high risk of bias, when information was lacking. When risk of bias could not be assessed, the item was classified as unclear. For the nonrandomized trials, the ROBINS-I tool was used [9]. Several sources of bias were assessed, such as confounding, selection of participants, classification of interventions, deviations from intended interventions, missing data, measurement of outcomes, selection of the reported result, and overall bias.

## 3. Results and Discussion

### 3.1. Main Characteristics of the Included Studies

**Figure 1:**
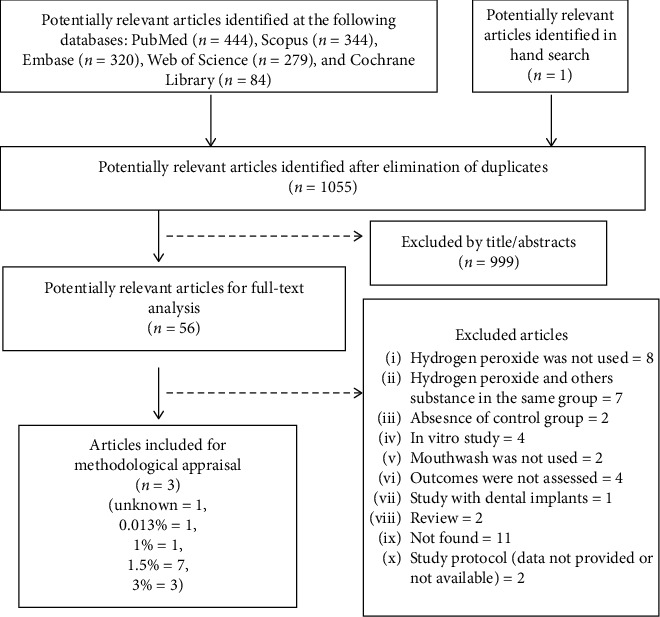
Flowchart of the studies during the review.

The search strategy and flowchart of articles retrieval is demonstrated in Figure 1. The reasons for exclusion of the identified studies are reported in Table S1. It should be noted that five databases were searched. The additional search strategies did not add any study to the present review, except for one additional study identified in the Google Scholar database [10]. The retrieved studies were very distinct in all aspects, preventing the possibility of a meta-analysis. Therefore, descriptive information will be given, according to design and outcome (experimental gingivitis or not; plaque, gingival inflammation, or microbiological parameters).

#### 3.1.1. Risk of Bias

**Figure 2:**
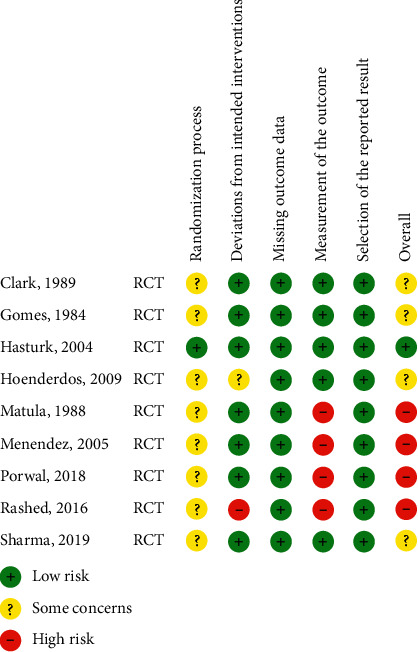
Risk of bias assessment for the randomized clinical trials.

Figure 2 demonstrates the analysis of risk bias of the randomized clinical trials included in this review according to the RoB2 instrument. It may be detected that only one study presented low risk of bias in all criteria analyzed [11]. Four other studies presented an overall high risk of bias [10, 12–14]. The criteria randomization process showed unclear risk of bias in almost all included studies. The other sources of bias comprised mainly lack of information of reproducibility.

The risk of bias for the nonrandomized trials included in the present review is demonstrated in Table 1. Bias due to confounding was critical in all studies. The other analyses mostly demonstrate moderate to low risk of bias.

#### 3.1.2. Plaque Index

*(1) Nonexperimental Gingivitis Studies*. The plaque index measurements are demonstrated in Table 2. It should be highlighted that 10 studies evaluated plaque parameters, of which six were performed as clinical trials allowing mechanical plaque control [11, 12, 15, 19–21]. In one, it was not possible to determine if mechanical plaque control was possible [10]. All studies that used H_2_O_2_ as adjunct to mechanical oral hygiene were performed with the concentration of 1.5%_._ Those studies demonstrate a higher antiplaque efficacy of chlorhexidine in comparison to H_2_O_2_, except two, in which H_2_O_2_ presented similar efficacy to chlorhexidine [10, 21]. Generally, very little differences from negative controls were detected.

*(2) Experimental Gingivitis Studies*. Among the studies that evaluated plaque parameters, 3 used the experimental gingivitis model [16, 18, 22]. The information coming from these studies gives an idea of efficacy of the mouth rinses in undisturbed dental biofilms. This enhances the proofs of principle of the antiplaque effect, which would give useful information, e.g., for areas where mechanical plaque control is not effective.

#### 3.1.3. Gingival Inflammation

*(1) Nonexperimental Gingivitis Studies*. The results related to gingival inflammatory parameters are presented in Table 3. Nine studies were included in this outcome [10–12, 15, 16, 18–21], six allowed mechanical plaque control [11, 12, 15, 19–21]. In one study, it was not clear if mechanical plaque control was allowed [10]. In these parameters, H_2_O_2_ mouth rinse performs better than negative controls, however less than chlorhexidine. A possibility of a decrease in inflammation could be raised since it seems that H_2_O_2_ performs better in terms of gingivitis than it does in relation to plaque.

*(2) Experimental Gingivitis Studies*. Among the studies that evaluated gingival inflammation, two were based on the experimental gingivitis model [16, 18]. The experimental gingivitis model provides information on the effect of the mouth rinse in areas in which plaque control is not adequate. Also, it rules out the eventual confounding effect of the adjunct plaque control in the study of chemical substances.

#### 3.1.4. Microbiological Outcomes

*(1) Nonexperimental Gingivitis Studies*. The results related to microbiological parameters are demonstrated in Table 4. Six studies were included with these outcomes [11, 13, 14, 16–18]. Four studies allowed mechanical control of biofilm [11, 13, 14, 17]. Better results with mouth rinses containing H_2_O_2_ when compared to a placebo were detected.

*(2) Experimental Gingivitis Studies*. Two studies performed microbiological analysis using an experimental gingivitis design [16, 18]. The information coming from such studies supports the quality/quantity of different germs when plaque is accumulating overtime. It also rules out the effect of the uncontrolled mechanical plaque removal.

#### 3.1.5. Qualitative Results—Safety

Among the 13 included studies, only five of them assessed for side effects. All of these five studies reported no side effects in individuals that used H_2_O_2_ mouthwashes [11, 14–16, 19]. Additionally, no side effects were reported in those that used chlorhexidine [14, 16]. Conversely, an increased tendency for desquamation of the mucosal lining was reported in individuals that used a placebo solution [16]. The other studies that used a negative control group reported no side effect in this group [11, 14, 15, 19].

### 3.2. Strengths and Limitations of SR

The present systematic review aimed to assess the eventual effects of mouth rinses with H_2_O_2_ on plaque, gingivitis, and different germs in the oral cavity. For that, five databases were searched, and 13 articles were methodologically appraised. In general, H_2_O_2_ mouth rinses demonstrated an effect on the three parameters under the study in different degrees.

Regarding the risk of bias of both randomized and nonrandomized clinical trials, it is important to highlight that most of the included studies presented an unclear or high risk of bias. Only one study demonstrated an overall low risk of bias [11]. This randomized clinical trial demonstrated a superior antigingivitis efficacy of H_2_O_2_ mouthwash in comparison to a placebo solution. However, no significant difference was observed for the antiplaque efficacy. The overall high risk of bias must be put into perspective when interpreting the results of the present study. This means that the use in clinical practice should be indicated with caution and not performed routinely since the support is not robust.

The strengths of the present systematic review were based on the importance of the topic, especially because the mouth is a very contaminated cavity and mouth rinses are used to reduce different degrees of contamination. In addition, with the COVID-19 pandemic, the use of mouth rinses has been considered an additional way for reducing all sorts of contamination. The limitations are related to the quality of the evidence. Therefore, the information contained herein should be cautiously interpreted. Also, in an attempt to decrease the time for publication of this information, no registration was performed and it was not possible to make a post hoc registration.

### 3.3. Quality of Evidence and Strength of Recommendation

Initially, the focused question included both a negative and a positive control group. The negative control could be either placebo, water, or no solution, whereas the control group should include the gold standard in terms of oral rinse—chlorhexidine. The results of this systematic review should be put into the perspective that H_2_O_2_ is widely used in oral care despite the lack of a large number of studies, especially in some of the aforementioned indications. We looked at the systematic review published by Hossainian et al. [3] that critically appraised the evidence until the beginning of this decade. Such work led to the conclusion that H_2_O_2_ does not consistently prevent plaque accumulation in short-term periods. Therefore, we expanded the search criteria, not restricting age, including microbiological parameters, updating the publication year to 2020, and including five databases instead of the two previously searched databases. Due to the higher usage of H_2_O_2_, we restricted the search to only include this substance and not any other oxygenating agent.

H_2_O_2_ has been used clinically for more than a century, and recently, H_2_O_2_ containing mouth rinse are being recommended, especially due to a possible antiviral effect and the pandemic of COVID-19. To the best of the authors' knowledge and making a systematic search in the same databases, no studies have observed any antiviral effect of H_2_O_2_ in the mouth. However, associations are supporting its use [5, 6]. The present systematic review used the most strict quality criteria for retrieving the studies. However, the interpretation will be contextualized in the moment that the world is facing a pandemic in which any kind of effort should be at least collated to make the sense of any preventive guideline.

In terms of plaque, one study [15] was performed in adolescents and the others in adults. One of them also included handicapped individuals [20]. Four of the six studies that allowed oral hygiene compared 1.5% H_2_O_2_ with a negative control [11, 15, 19, 20] and 2 of them with chlorhexidine [12, 21], and one of them was also compared to a negative control [12]. Among the studies that used the experimental gingivitis model [16, 18, 22], two were compared with a negative control [18, 22] and the other included a positive control [16]. In one study, the effect of H_2_O_2_ was compared to chlorhexidine, but it was not possible to determine if mechanical plaque control was allowed [10]. In these studies, different concentrations of H_2_O_2_ were used.

It is clear from the encountered results that 1.5% H_2_O_2_ is the most studied concentration in the formula of a mouth rinse. This result is in accordance with the previously published review [3]. For the publications evaluating the effect of H_2_O_2_ on plaque, only one study (which evaluated the antiplaque effect over an 18-month time period) demonstrated improved results when compared to a placebo [15]. The other studies, which evaluated the effect over shorter periods, did not find statistically significant differences. Also, in the studies that used the experimental gingivitis model, only one study demonstrated the superiority of H_2_O_2_ in comparison to placebo [18].

The same cited publications that evaluated plaque also evaluated the effect of H_2_O_2_ on gingival inflammation. Although only a single study demonstrated the antiplaque benefit of H_2_O_2_, more studies clearly point to a better antigingivitis effect of H_2_O_2_ mouth rinses as compared to placebo [11, 15, 20]. In fact, for one of the studies, no difference was observed between the H_2_O_2_ mouth rinse and the positive control [12]. Because the participants of these studies were allowed for routine mechanical oral hygiene, an effect on clinical inflammation alone (without having the associated plaque reduction benefit) should be highlighted. These results suggest that H_2_O_2_ might perform differently in terms of plaque and gingivitis, which is of great clinical interest.

Also, it is of high importance to evaluate the effect of mouth rinses on the oral microbiome. This includes not only bacteria but also other germs, such as viruses and fungi. However, despite completing a broad search of the literature, no studies were identified that evaluated the effect of H_2_O_2_ oral microorganisms other than bacteria. The comparisons of the effect of rinses on oral bacteria with H_2_O_2_ and with the positive control generally demonstrate a better effect of the latter. However, the differences in terms of the composition of the oral microbiome when H_2_O_2_ is compared to placebo are clear in a variety of bacterial species. The present study evaluated risk of bias both for the nonrandomized and randomized trials. As expected, the randomized clinical trials presented a higher quality, with decreased risk of bias. The nonrandomized studies in general present a higher risk of bias. This is inherent to the chosen design. Randomized studies tend to present a lower risk of bias.

A systematic review was recently published by Marui et al. [23] describing the effect of preprocedural rinses with different substances on dental office-generated aerosols. They demonstrated that rinses with chlorhexidine, essential oils, and cetylpyridinium chloride are effective. No studies with H_2_O_2_ were included.

### 3.4. Implications for Further Research

Meanwhile, taking into consideration the precautionary principle [24], even without the qualified evidence, due to the high levels of morbimortality, it is of interest to see other potentials of the use of H_2_O_2_. In such conditions, the use of “collateral evidence” is recommended, always with a surveillance look. Therefore, in the present moment, further studies including oral rinses with H_2_O_2_ and other substances are warranted. Studies with the antiviral effect of H_2_O_2_ are also needed.

## 4. Conclusions

In conclusion, rinsing with 1.5% H_2_O_2_ has demonstrated an antigingivitis effect as compared to placebo, with also greater reductions in oral bacteria. Chlorhexidine has demonstrated, up to now, the best antiplaque and antigingivitis effect on the oral microbiome.

## Figures and Tables

**Table 1:** Risk of bias of the nonrandomized clinical trial, assessed by ROBINS-I tool, included in the present systematic review.

Author, year	Bias due to confounding	Bias in selection of participants into the study	Bias in classification of interventions	Bias due to deviations from intended interventions	Bias due to missing data	Bias in measurement of outcomes	Bias in selection of the reported result	Overall bias
Boyd, 1989 [15]	Critical	Low	Moderate	Low	Moderate	Moderate	Low	Moderate

Gusberti, 1988 [16]	Critical	Low	Moderate	Low	Low	Serious	Low	Critical

Pitten, 1999 [17]	Critical	Low	Moderate	Moderate	Low	Serious	Low	Critical

Wennström, 1979 [18]	Critical	Low	Moderate	Low	Low	Moderate	Low	Serious

**Table 2:** Main characteristics and results for the studies that assess plaque index.

Study, year, country (reference) Study design	Systemic conditions Diagnosis of the oral condition Plaque index assessed	H_2_O_2_ group (concentration) Rinsing protocol N (smokers) Age Baseline N(M)/N(F) End N(M)/N(F)	CHX group (concentration) Rinsing protocol N (smokers) Age Baseline N(M)/N(F) End N(M)/N(F)	Control group Rinsing protocol N (smokers) Age Baseline N(M)/N(F) End N(M)/N(F)	Main results (follow-up)			
					Baseline scores Mean ± SD	End scores (follow-up) Mean ± SD	∆ (%)	Author conclusions
*Studies without experimental gingivitis*								

Boyd, 1989, USA [15]; Non-RCT	Systemically healthy; Adolescents with initial oral health scheduled to have fixed orthodontic treatment; PI (S & L)	H_2_O_2_ 1.5% + 0.05% NaF + toothbrush; Once-a-day (1/2 ounce per one minute) during 18 months; Smokers NR; Mean age:13.2 yrs; Baseline: 9/17; End: 24 individuals in total (proportion of M/F NR).	NA	0.05% NaF + toothbrush; Once-a-day (1/2 ounce per one minute) during 18 months; Smokers NR; Mean: 13.5 yrs; Baseline: 11/23; End: 32 individuals in total; (proportion of M/F NR).	Percent of sites with PI (S & L) >1: A:13.15 ± 17.40 B: NA C: 19.05 ± 26.00	(18 months) Percent of sites with PI (S & L) >1: A: 18.50 ± 13.18 B: NA C: 36.79 ± 35.60	PI (S & L) A: 5.35 (40.68%) B: NA C: 17.74 (93.12%^#^)^*∗∗*^	When the percentage of sites with plaque index greater than 1 was considered, the group that used H_2_O_2_ showed significantly lower percentage when compared to the control group

Clark, 1989, USA [19] RCT (parallel)	Systemically healthy; Gingivitis (at least 8 sites with marginal gingival bleeding) PIS (Q & H)	H_2_O_2_ 1.5% (use of toothbrush and dentifrice NR); Once-a-day (20 ml for 30 s) during 21 weeks; 2 smokers Mean age: 28.9 yrs Baseline: 9/16 End: 22 individuals in total (proportion of M/F NR)	NA	Water (use of toothbrush and dentifrice NR); Once-a-day (20 ml for 30 s) during 21 weeks; No smokers Mean age 27.8 yrs Baseline: 8/17 End: 22 individuals in total (proportion of M/F NR)	A: 2.87 ± 0.38 B: NA C: 2.90 ± 0.25	(6 months) A: 1.72 ± 0.58 B: NA C: 1.74 ± 0.46	A: −1.15 (−40.07%) B: NA C: −1.16 (−40.00%)	No statistically significant difference between groups

Gomes, 1984, USA [20] RCT (parallel)	Nonhandicapped staff and handicapped patients—both of them were free of systemic diseases Individuals without periodontitis (probing depth <5 mm) PI (L)	H_2_O_2_ 1.5% (use of toothbrush and dentifrice NR) Three times per day (10 ml per 60 s) during 7 days Staff: smokers NR Mean age: 30.34 yrs Baseline: 5/34 End: NR/NR Patients: smokers NR Mean age: 28.65 yrs Baseline: 30/24 End: NR/NR	NA	Placebo mouth rinse (use of toothbrush and dentifrice NR) Three times per day (10 ml per 60 s) during 7 days Staff: smokers NR Mean age: 29.94 yrs Baseline: 5/30 End: NR/NR Patients: smokers NR Mean age: 28.62 yrs Baseline: 25/25 End: NR/NR	Staff—male A: 0.68 ± 0.54 B: NA C: 0.66 ± 0.38 Staff—female A: 0.61 ± 0.50 B: NA C: 0.43 ± 0.24 Patients—male A: 1.29 ± 0.55 B: NA C: 1.53 ± 0.69 Patients—female A: 1.30 ± 0.55 B: NA C: 1.23 ± 0.59	(7 days) Staff—male A: 0.54 ± 0.49 B: NA C: 0.72 ± 0.52 Staff—female A: 0.45 ± 0.50 B: NA C: 0.41 ± 0.26 Patients—male A: 1.05 ± 0.55 B: NA C: 1.51 ± 0.55 Patients—female A: 1.07 ± 0.41 B: NA C: 1.19 ± 0.57	Staff—male A: −0.14 (−20.59%^#^) B: NA C: 0.06 (9.09%) Staff—female A: −0.16 (−26.22%^#^) B: NA C: −0.02 (−4.65%) Patients—male A: −0.24 (−18.60%^#^) B: NA C: −0.02 (−1.31%) Patients—female A: −0.23 (−17.69%^#^) B: NA C: −0.04 (−3.25%)	In the within group comparison, a significantly lower plaque score was detected, for both staff and patient groups, only for H_2_O_2_
Hasturk, 2004; USA [11] RCT (parallel)	Systemically healthy Gingivitis (gingival index >2.0 and >10% sites with bleeding). In the first 28 days, only the mouth rinses were used. PIS (Q & H)	H_2_O_2_ 1.5% + 0.05 sodium fluoride toothpaste + toothbrush Twice daily (quantity not reported for 30 s) during 6 months; 3.9% smokers (N not provided) Mean age: 31.4 yrs; Baseline: 19%/51% (N not provided) End: NR/NR	NA	Placebo mouth rinse 0.05 sodium fluoride toothpaste + toothbrush Twice daily (quantity not reported for 30 s) during 6 months 18.8% smokers (N not provided) Mean age: 31.0 yrs Baseline: 45.8%/54.2% (N not provided) End: NR/NR	A: 1.03 ± 0.81 B: NA C: 0.87 ± 0.58	(6 months) A: 0.99 ± NR B: NA C: 0.99 ± NR	A: −0.04 (−3.88%) B: NA C: 0.12 (13.79%)	No statistically significant difference between groups

Porwal, 2018, India [10] RCT (parallel)	Systemically healthy Gingivitis (modified gingival index of 3 and plaque index of 4) PIS (Q & H)	H_2_O_2_ 3% with distilled water (ratio 1 : 1) Twice daily (10 ml and time not reported) during 15 days Smokers NR Mean age: NR Baseline: 10 individuals in total (proportion of M/F NR) End: 10 individuals in total (proportion of M/F NR)	CHX 0.2% with distilled water (ratio 1 : 1) Twice daily (10 ml and time not reported) during 15 days Smokers NR Mean age: NR Baseline: 10 individuals in total (proportion of M/F NR) End: 10 individuals in total (proportion of M/F NR)	NA	A: 3.12 ± 0.44 B: 3.26 ± 0.53 C: NA	(28 days) A: 1.28 ± 0.86 B: 0.82 ± 0.54 C: NA	A: −1.84 (−58.97%^#^) B: −2.44 (−74.85%^#^) C: NA	No statistically significant difference between groups

Rashed, 2016, Saudi Arabia [12] RCT (parallel)	Systemically healthy Periodontitis (clinical attachment loss >4 mm in >30% of the sites) PI (O'L)	H_2_O_2_ 1.5% + scaling and root planning + mechanical oral hygiene Twice daily (quantity and time not reported) during 10 days No smokers Age range: 30–50 yrs Baseline: 15/0 End: 15/0	CHX 0.2% + scaling and root planing + mechanical oral hygiene Twice daily (quantity and time not reported) during 10 days No smokers Age range: 30−50 yrs Baseline: 15/0 End: 15/0	Scaling and root planing only + mechanical oral hygiene Not applicable No smokers Age range: 30−50 Baseline: 15/0 End: 15/0	A: NR ± NR B: NR ± NR C: NR ± NR	(3 months) A: NR ± NR B: NR ± NR C: NR ± NR	A: NR (NR^#^) B: NR (NR^#^) C: NR (NR^#^)	No statistically significant difference among groups

Sharma, 2019; India [21] RCT (parallel)	Systemically healthy; Halitosis (patients with organoleptic score >1, in a scale of 0 to 5); PI (S & L)	H_2_O_2_ 1.5% (use of toothbrush and dentifrice NR) Twice daily (10 ml and time not reported) during 15 days No smokers Mean age: 24.78 yrs (whole-sample) Baseline: NR/NR End: NR/NR	CHX 2% (use of toothbrush and dentifrice NR) Twice daily (10 ml and time not reported) during 15 days No smokers Mean age: 24.78 yrs (whole-sample) Baseline: NR/NR End: NR/NR	NA	A: 1.82 ± 0.13 B: 1.78 ± 0.22 C: NA	(15 days) A: 1.68 ± 0.12 B: 1.50 ± 0.26 C: NA	A: −0.14 (−7.69%^#^) B: −0.28 (−15.73%^#^)^*∗*^ C: NA	CHX group showed significantly lower plaque in comparison to H_2_O_2_

*Studies with experimental gingivitis*								
Gusberti, 1988, Switzerland [16] Non-RCT (parallel)	Not reported Not reported PI (S & L)	H_2_O_2_ 1% Twice daily (15 ml for 30 s) during 21 days Smokers NR Mean age NR Baseline: NR/NR End: NR/NR	CHX 0.12% Twice daily (15 ml for 30 s) during 21 days Smokers NR Mean age NR Baseline: NR/NR End: NR/NR	Flavored alcoholic placebo solution Twice daily (15 ml for 30 s) Smokers NR Mean age NR Baseline: NR/NR End: NR/NR	A: 0.00 ± 0.00 B: 0.00 ± 0.00 C: 0.00 ± 0.00	(21 days) A: 1.40 ± NR B: 0.35 ± NR C: 1.56 ± NR	A: 1.40 B: 0.35^*∗*^ C: 1.56	Significantly lower plaque index detected in the CHX group when compared to both groups No statistically significant difference was detected between H_2_O_2_ and control groups

Hoenderdos, 2009 [22] Netherlands RCT (parallel)	Systemically healthy Periodontally healthy (no sites with probing depth >5mm) PIS (Q & H)	H_2_O_2_ 0.013% + 0.004% glycerol dissolved in demineralized water Twice daily (15–20 ml for 20 s) during 3 days Smokers NR Mean: 24.5 yrs (whole-sample) Baseline: NR/NR End: NR/NR	NA	0.004% glycerol dissolved in demineralized water Twice daily (15–20 ml for 20 s) during 3 days Smokers NR Mean: 24.5 yrs (whole-sample) Baseline: NR/NR End: NR/NR	A: 0.00 ± 0.00 B: NA C: 0.00 ± 0.00	(3 days) A: 2.66 ± 0.29 B: NA C: 2.70 ± 0.32	A: 2.66 B: NA C: 2.70	No statistically significant difference was detected between groups

Wennström, 1979; Sweden [18] Non-RCT (cross-over)	Systemically healthy Oral health PI (S & L)	H_2_O_2_ (1.7 g dissolved in 30 ml of tap water) Three times a day (30 ml per 2 minutes) during 14 days Smokers NR Mean age NR Baseline: NR/NR (total *n* = 14) End: NR/NR (total *n* = 14).	NA	Placebo mouthwash Three times a day (30 ml per 2 minutes) during 14 days Smokers NR Mean age NR Baseline: NR/NR (total *n* = 14) End: NR/NR (total *n* = 14).	Percent of sites with score 0 A: 98.90 ± 0.50 B: NA C: 98.70 ± 0.50 Percent of sites with score 1 A: 1.00 ± 0.40 B: NA C: 1.30 ± 0.50 Percent of sites with score 2 A: 0.10 ± 0.10 B: NA C: 0.00 ± 0.00 Percent of sites with score 3 A: 0.00 ± 0.00 B: NA C: 0.00 ± 0.00	(14 days) Percent of sites with score 0 A: 46.40 ± 4.30 B: NA C: 17.20 ± 2.90 Percent of sites with score 1 A: 45.20 ± 3.60 B: NA C: 37.00 ± 4.80 Percent of sites with score 2 A: 8.70 ± 3.60 B: NA C: 44.40 ± 6.20 Percent of sites with score 3 A: 0.00 ± 0.00 B: NA C: 1.40 ± 0.90	Sites with score 0 A: −52.50 (−53.08%) B: NA C: −81.50 (−82.57%)^*∗∗*^ Sites with score 1 A: 44.20 (4420%) B: NA C: 35.70 (2,746%) Sites with score 2 A: 8.6 (8,600%) B: NA C: 44.4^*∗∗*^ Sites with score 3 A: 0 (0%) B: NA C: 1.40^*∗∗*^	Significantly higher percentage of score 0 was detected in the H_2_O_2_ group in comparison to the control group No statistically significant difference was detected between groups of score 1 Significantly lower percentage of score 2 was detected in the H_2_O_2_ group in comparison to the control group Significantly lower percentage of score 2 was detected in the H_2_O_2_ group in comparison to the control group

NA: not applicable; NR: not reported; RCT: randomized clinical trial; non-RCT: nonrandomized clinical trial; CHX: chlorhexidine. M: male; F: female. A: H_2_O_2_ rinse; B: chlorhexidine rinse; C: control rinse. ∆: delta of mean (final−initial mean); %: percentage of reduction. PI (S & L): plaque index (Silness & Löe, 1964); PIS (Q & H): Quigley–Hein plaque modified by Turesky plaque index; PI (L): plaque index (Löe, 1967); PI (O'L): O'Leary plaque index. ^*∗*^Statistically significant differences between groups (H_2_O_2_ vs chlorhexidine); ^*∗∗*^statistically significant differences between groups (H_2_O_2_ vs control); ^#^statistically significant differences within group (end vs baseline score).

**Table 3:** Main characteristics and results for the studies that assess gingival inflammation.

Study, year, country (reference) Study design	Systemic conditions; Diagnosis of the oral condition Gingival index assessed	H_2_O_2_ group (concentration); Rinsing protocol N (smokers) Age Baseline; N(M)/N(F) End N(M)/N(F)	CHX group (concentration); Rinsing protocol N (smokers); Age Baseline; N(M)/N(F) End N(M)/N(F)	Control group Rinsing protocol N (smokers) Age Baseline; N(M)/N(F) End N(M)/N(F)	Main results (follow-up)			
					Baseline scores Mean ± SD	End scores (follow-up) Mean ± SD	∆ (%)	Author conclusions
*Studies without experimental gingivitis*								
Boyd, 1989, USA [15] Non-RCT	Systemically healthy Adolescents with initial good oral health scheduled to have fixed orthodontic treatment GI (L & S) and BT (A)	H_2_O_2_ 1.5% + 0.05% NaF + toothbrush Once a day (1/2 ounce per one minute) during 18 months Smokers NR Mean age:13.2 yrs Baseline: 9/17 End: 24 individuals in total (proportion of M/F NR)	NA	0.05% NaF + toothbrush Once a day (1/2 ounce per one minute) during 18 months Smokers NR Mean: 13.5 yrs Baseline: 11/23 End: 32 individuals in total (proportion of M/F NR)	Percent of sites with GI (L & S) >1 A: 17.41 ± 20.74 B: NA C: 21.61 ± 24.93 Percent of sites with BT(A) >1: A: 17.69 ± 21.77 B: NA C: 22.70 ± 25.24	(18 months) Percent of sites with GI (L & S) >1 A: 27.04 ± 24.69 B: NA C: 53.46 ± 29.38 Percent of sites with BT (A) >1: A: 24.52 ± 25.77 B: NA C: 53.19 ± 30.49	GI (L & S) A: 9.63 (55.31%) B: NA C: 31.85 (147.39%^#^)^*∗∗*^ BT (A) A: 6.83 (38.61%) B: NA C: 30.49 (134.32%^#^)^*∗∗*^	When the percentage of sites with gingival index >1 was considered, the group that used H_2_O_2_ showed significantly lower percentage when compared to the placebo group When the percentage of sites with bleeding tendency >1 was considered, the group that used H_2_O_2_ showed significantly lower percentage when compared to the placebo group

Clark, 1989, USA [19] RCT (parallel)	Systemically healthy Gingivitis (at least 8 sites with marginal gingival bleeding) GI (L & S)	H_2_O_2_ 1.5% (use of toothbrush and dentifrice NR) Once a day (20 ml for 30 s) during 21 weeks 2 smokers Mean age: 28.9 yrs Baseline: 9/16 End: 22 individuals in total (proportion of M/F NR)	NA	Water (use of toothbrush and dentifrice NR) Once a day (20 ml for 30 s) during 21 weeks No smokers Mean age 27.8 yrs Baseline: 8/17 End: 22 individuals in total (proportion of M/F NR)	A: 2.20 ± 0.35 B: NA C: 2.31 ± 0.24	(6 months) A: 1.62 ± 0.61 B: NA C: 1.88 ± 0.50	A: −0.58 (−26.367%) B: NA C: −0.43 (−18.61%)	No statistically significant difference between groups
Gomes, 1984, USA [20] RCT (parallel)	Nonhandicapped staff and handicapped patients—both of them were free of systemic diseases Individuals without periodontitis (probing depth <5 mm) GI (L & S)	H_2_O_2_ 1.5% (use of toothbrush and dentifrice NR) Three times per day (10 ml per 60 s) during 7 days Staff: Smokers NR Mean age: 30.34 yrs Baseline: 5/34 End: NR/NR Patients: smokers NR Mean age: 28.65 yrs Baseline: 30/24 End: NR/NR	NA	Placebo mouth rinse (use of toothbrush and dentifrice NR) Three times per day (10 ml per 60 s) during 7 days Staff: Smokers NR Mean age: 29.94 yrs Baseline: 5/30 End: NR/NR Patients: Smokers NR Mean age: 28.62 yrs Baseline: 25/25 End: NR/NR	Staff—male A: 1.28 ± 0.58 B: NA C: 1.00 ± 0.12 Staff—female A: 1.30 ± 0.46 B: NA C: 0.89 ± 0.31 Patients—male A: 1.37 ± 0.48 B: NA C: 1.64 ± 0.63 Patients—female A: 1.36 ± 0.48 B: NA C: 1.31 ± 0.59	(7 days) Staff—male A: 0.73 ± 0.29 B: NA C: 1.01 ± 0.32 Staff—female A: 0.85 ± 0.41 B: NA C: 0.86 ± 0.32 Patients—male A: 1.19 ± 0.49 B: NA C: 1.58 ± 0.58 Patients—female A: 1.18 ± 0.42 B: NA C: 1.25 ± 0.55	Staff—male A: −0.55 (−42.97%^#^) B: NA C: 0.01 (1.00%) Staff—female A: −0.45 (−34.62%^#^) B: NA C: −0.03 (−3.37%) Patients—male A: −0.18 (−13.14%^#^) B: NA C: −0.06 (−3.66%) Patients—female A: −0.18 (−13.24%^#^) B: NA C: −0.06 (−4.58%)	In the within group comparison, a significantly lower plaque score was detected, for both staff and patient groups, only for H_2_O_2_

Hasturk, 2004; USA [11] RCT (parallel)	Systemically healthy Gingivitis (gingival index >2.0 and >10% sites with bleeding) In the first 28 days, only the mouth rinses were used EIBI; mGI (G) and BoP	H_2_O_2_ 1.5% + 0.05 sodium fluoride toothpaste + toothbrush Twice daily (quantity not reported for 30 s) during 6 months 3.9% smokers (N not provided) Mean age: 31.4 yrs Baseline: 19%/51% (N not provided) End: NR/NR	NA	Placebo mouth rinse 0.05 sodium fluoride toothpaste + toothbrush Twice daily (quantity not reported for 30 s) during 6 months 18.8% smokers (N not provided) Mean age: 31.0 yrs Baseline: 45.8%/54.2% (N not provided) End: NR/NR	EIBI A: 0.05 ± 0.14 B: NA C: 0.04 ± 0.09 mGI (G) A: 1.81 ± 0.41 B: NA C: 1.79 ± 0.46 BoP A: 0.12 ± 0.17 B: NA C: 0.10 ± 0.12	(6 months) EIBI A: 0.02 ± NR B: NA C: 0.02 ± NR mGI (G) A: 1.63 ± NR B: NA C: 1.83 ± NR BoP A: 0.08 ± NR B: NA C: 0.10 ± NR	EIBI A: −0.03 (−64.34%^#^) B: NA C: −0.02 (−38.42%^#^) mGI (G) A: −0.18 (−9.95%^#^) B: NA C: 0.04 (2.23%)^*∗∗*^ BoP A: −0.02 (−20%) B: NA C: −0.02 (−16.81%)	The decrease in the H_2_O_2_ group was significant in comparison to change in the placebo group (*P* = 0.004) when considering the mGI (G) No other statistically significant difference between groups was observed
Porwal, 2018, India [10] RCT (parallel)	Systemically healthy Gingivitis (modified gingival index of 3 and plaque index of 4) mGI (L)	H_2_O_2_ 3% with distilled water (ratio 1:1) Twice daily (10 ml and time not reported) during 15 days Smokers NR Mean age: NR Baseline: 10 individuals in total (proportion of M/F NR) End: 10 individuals in total (proportion of M/F NR)	CHX 0.2% with distilled water (ratio 1:1) Twice daily (10 ml and time not reported) during 15 days Smokers NR Mean age: NR Baseline: 10 individuals in total (proportion of M/F NR) End: 10 individuals in total (proportion of M/F NR)	NA	A: 2.92 ± 0.31 B: 3.04 ± 0.23 C: NA	(28 days) A: 0.86 ± 0.11 B: 0.54 ± 0.35 C: NA	A: −2.06 (−70.55%^#^) B: −2.50 (−82.24%^#^) C: NA	No statistically significant difference between groups

Rashed, 2016, Saudi Arabia [12] RCT (parallel)	Systemically healthy Periodontitis (clinical attachment loss >4 mm in >30% of the sites) GI (L & S)	H_2_O_2_ 1.5% + scaling and root planing + mechanical oral hygiene Twice daily (quantity and time not reported) during 10 days No smokers Age range: 30–50 yrs Baseline: 15/0 End: 15/0	CHX 0.2% + scaling and root planing + mechanical oral hygiene Twice daily (quantity and time not reported) during 10 days No smokers Age range: 30–50 yrs Baseline: 15/0 End: 15/0	Scaling and root planing only + mechanical oral hygiene Not applicable No smokers Age range: 30–50 Baseline: 15/0 End: 15/0	A: NR ± NR B: NR ± NR C: NR ± NR	(3 months) A: NR ± NR B: NR ± NR C: NR ± NR	A: NR (NR^#^) B: NR (NR^#^) C: NR (NR^#^)^*∗∗*^	No statistically significant difference between CHX and H_2_O_2_ groups Statistically significant lower gingival index in H_2_O_2_ in comparison to the control group

Sharma, 2019; India [21] RCT (parallel)	Systemically healthy Halitosis (patients with organoleptic score >1, in a scale of 0 to 5) GI (L & S)	H_2_O_2_ 1.5% (use of toothbrush and dentifrice NR) Twice daily (10 ml and time not reported) during 15 days No smokers Mean age: 24.78 yrs (whole sample) Baseline: NR/NR End: NR/NR	CHX 2% (use of toothbrush and dentifrice NR) Twice daily (10 ml and time not reported) during 15 days No smokers Mean age: 24.78 yrs (whole sample) Baseline: NR/NR End: NR/NR	NA	A: 1.83 ± 0.35 B: 1.74 ± 0.17 C: NA	(15 days) A: 1.80 ± 0.34 B: 1.66 ± 0.17 C: NA	A: −0.03 (−1.64%^#^) B: −0.08 (−4.60%^#^)^*∗*^ C: NA	CHX group showed significantly lower gingival index scores in comparison to H_2_O_2_
*Studies with experimental gingivitis*								
Gusberti, 1988, Switzerland [16] Non-RCT (parallel)	Not reported Not reported GI (L & S)	H_2_O_2_ 1% Twice daily (15 ml for 30 s) during 21 days Smokers NR Mean age NR Baseline: NR/NR End: NR/NR	CHX 0.12% Twice daily (15 ml for 30 s) during 21 days Smokers NR Mean age NR Baseline: NR/NR End: NR/NR	Flavored alcoholic placebo solution Twice daily (15 ml for 30 s) Smokers NR Mean age NR Baseline: NR/NR End: NR/NR	A: 0.00 ± 0.00 B: 0.00 ± 0.00 C: 0.00 ± 0.00	(21 days) A: 1.27 ± NR B: 0.06 ± NR C: 1.49 ± NR	A: 1.27 B: 0.06^*∗*^ C: 1.49^*∗∗*^	Statistically significant lower gingival index was detected in the CHX group when compared to both groups Statistically significant lower gingival index was detected in the H_2_O_2_ group when compared to the placebo group

Wennström, 1979, Sweden [18] Non-RCT (cross-over)	Systemically healthy Oral health GI (L & S)	H_2_O_2_ (1.7g dissolved in 30 ml of tap water) Three times a day (30 ml per 2 minutes) during 14 days Smokers NR Mean age NR Baseline: NR/NR (total *n* = 14) End: NR/NR (total *n* = 14)	NA	Placebo mouthwash Three times a day (30 ml per 2 minutes) during 14 days Smokers NR Mean age NR Baseline: NR/NR (total *n* = 14); End: NR/NR (total *n* = 14)	Percent of sites with score 0 A: 72.10 ± 1.80 B: NA C: 74.00 ± 2.30 Percent of sites with score 1 A: 26.4 ± 1.70 B: NA C: 25.4 ± 2.20 Percent of sites with score 2 A: 1.50 ± 0.60 B: NA C: 0.60 ± 0.30 Percent of sites with score 3 A: 0.00 ± 0.00 B: NA C: 0.00 ± 0.00	(14 days) Percent of sites with score 0 A: 27.40 ± 7.30 B: NA C: 13.00 ± 2.30 Percent of sites with score 1 A: 53.30 ± 1.40 B: NA C: 54.90 ± 3.40 Percent of sites with score 2 A: 19.40 ± 2.40 B: NA C: 31.60 ± 4.60 Percent of sites with score 3 A: 0.00 ± 0.00 B: NA C: 0.00 ± 0.00	Sites with score 0 A: −44.70 (−62.00%) B: NA C: −61.00 (−82.43%)^*∗∗*^ Sites with score 1 A: 26.90 (101.89%) B: NA C: 29.50 (116.14%) Sites with score 2 A: 17.9 (1.193%) B: NA C: 31 (5,166%)^*∗∗*^ Sites with score 3 A: 0 (0%) B: NA C: 0 (0%)	Significantly higher percentage of score 0 was detected in the H_2_O_2_ group in comparison to the control group Significantly a lower percentage of score 1 was detected in the H_2_O_2_ group in comparison to the control group Significantly a lower percentage of score 2 was detected in the H_2_O_2_ group in comparison to the control group No statistically significant difference was detected between groups for percentage of score 3

NA: not applicable; NR: not reported; RCT: randomized clinical trial; non-RCT: nonrandomized clinical trial; CHX: chlorhexidine; M: male; F: female. A: H_2_O_2_ rinse; B: chlorhexidine rinse; C: control rinse. ∆: delta of mean (final−initial mean); %: percentage of reduction. GI (L & S): gingival index (Löe & Silness, 1963); BT (A): bleeding tendency (Armitage et al., 1982); EIBI: Eastman interdental bleeding index; mGI (G); modified gingival index (Gordon et al., 1985); BoP: bleeding on probing; mGI (L): modified gingival index (Lobene et al., 1989). ^*∗*^Statistically significant differences between groups (H_2_O_2_ vs chlorhexidine); ^*∗∗*^statistically significant differences between groups (H_2_O_2_ vs control); ^#^statistically significant differences within group (end vs baseline score).

**Table 4:** Main characteristics and results for the studies that performed microbiological analysis.

Study, year, country (reference) Study design	Systemic conditions Diagnosis of the oral condition Microbiological analysis performed	H_2_O_2_ group (concentration) Rinsing protocol N (smokers) Age Baseline N(M)/N(F) End N(M)/N(F)	Chlorhexidine group (concentration), rinsing protocol N (smokers) Age Baseline N(M)/N(F) End N(M)/N(F)	Control group Rinsing protocol N (smokers) Mean age Baseline N(M)/N(F) End N(M)/N(F)	Main results			
					Baseline scores Mean ± SD	End scores (follow-up) Mean ± SD	∆ (%)	Author conclusions
*Studies without experimental gingivitis*								
Hasturk, 2004; USA [11] RCT (parallel)	Systemically healthy Gingivitis (gingival index >2.0 and >10% sites with bleeding). In the first 28 days, only the mouth rinses were used. *F. nucleatum ss*. *vincenti*, *C. concisus*, *C. rectus*, *T. forsythensis*, *P. gingivalis*, *P. intermedia*, *P. nigrescens*, *C. sputigena*, *S. oralis*, *A. naeslundii*, *T. denticola, C. curva,* and *E. corrodens*	H_2_O_2_ 1.5% + 0.05 sodium fluoride toothpaste + toothbrush Twice daily (quantity not reported for 30 s) during 6 months 3.9% smokers (N not provided) Mean age: 31.4 yrs Baseline: 19%/51% (N not provided) End: NR/NR	Not applicable	Placebo mouth rinse 0.05 sodium fluoride toothpaste + toothbrush Twice daily (quantity not reported for 30 s) during 6 months 18.8% smokers (N not provided) Mean age: 31.0 yrs Baseline: 45.8%/54.2% (N not provided) End: NR/NR	A: NR B: NA C: NR	(6 months) A: NR B: NA C: NR	A: NR B: NA C: NR	Various degrees of reductions in *F. nucleatum ss*. *vincentii*, *C. concisus*, *C. rectus*, *T. forsythensis*, *P. gingivalis*, *P nigrescens*, *C. sputigina,* and *E. corrodens*, with the use of the test rinse but not the placebo None of these changes, however, were statistically significant

Matula, 1988, Austria [13] RCT (cross-over)	Systemically healthy Not reported Total anaerobic/aerobic microbial counts	H_2_O_2_ 3% aqueous solution Single usage (12 ml for 60 s) Smokers NR Mean age: 23–49 yrs Baseline: NR/NR (total *n* = 12) End: NR/NR (total *n* = 12)	Not applicable	Water Single usage (12 ml for 60 s) Smokers NR Mean age: 23–49 yrs Baseline: NR/NR (total *n* = 12) End: NR/NR (total *n* = 12)	Mean aerobic bacteria A: 100% B: NA C: 100% Mean anaerobic bactéria A: 100% B: NA C: 100%	(60 minutes) Mean aerobic bacteria A: 49.60% ± NR B: NA C: 103.10% ± NR Mean anaerobic bactéria A: 41.40% ± NR B: NA C: 112.00% ± NR	Mean aerobic bacteria A: 50.40% B: NA C: 3.10%^*∗∗*^ Mean anaerobic bactéria A: 58.60% B: NA C: 12^*∗∗*^	H_2_O_2_ presented higher reductions in both aerobic and anaerobic bacteria as compared to control, except for aerobic bacteria at 30 minutes

Menendez, 2005, USA [14] RCT (cross-over)	Systemically healthy Not reported Total Streptococci levels and *S. mutans* levels in saliva	H_2_O_2_ 1.5% Twice daily (15 ml for 60s) during 21 days Smokers NR Mean age: 26–55 yrs Baseline: NR/NR (total *n* = 16) End: NR/NR (total *n* = 16)	CHX 0.12% Twice daily (15 ml for 60 s) during 21 days Smokers NR Mean age: 26–55 yrs Baseline: NR/NR (total *n* = 16) End: NR/NR (total *n* = 16)	Placebo Twice daily (15 ml for 60 s) during 21 days Smokers NR Mean age: 26–55 yrs Baseline: NR/NR (total *n* = 16) End: NR/NR (total *n* = 16)	Total Streptococci A: NR B: NR C: NR *S. mutans* (in CFU/ml) A: NR B: NR C: NR	(21 days) Total Streptococci A: 885857 ± 218478 B: 121465 ± 273913 C: 908645 ± 211957 *S. mutans* (in CFU/ml) A: 10442 ± 7845 B: 11614 ± 7685 C: 1696 ± 1933	Total Streptococci A: NR B: NR C: NR *S. mutans* (in CFU/ml) A: NR B: NR C: NR	Chlorhexidine performed better in total level of Streptococcus, as compared to H_2_O_2_, that did not differ from placebo No statistically significant differences were observed in *S. mutans* among groups

Pitten, 1999, Germany [17] Non-RCT	Systemically healthy Not reported Reduction factors of log CFU	H_2_O_2_ 3% Single usage (20 ml, 30 seconds, followed by 20 ml of sterile water for additional 30 seconds) Smokers NR Mean age: NR Baseline: NR/NR (total *n* = at least 10) End: NR/NR (total *n* = at least 10)	CHX 0.2% Single usage (20 ml, 30 seconds, followed by 20 ml of sterile water for additional 30 seconds) Smokers NR Mean age: NR Baseline: NR/NR (total *n* = at least 10) End: NR/NR (total *n* = at least 10)	Distilled sterile water Single usage (20 ml, 30 seconds, followed by another 20 ml of sterile water for additional 30 seconds) Smokers NR Mean age: NR Baseline: NR/NR (total *n* = at least 10) End: NR/NR (total *n* = at least 10	A: NR B: NR C: NR	(60 minutes) A: 0.35 ± NR B: 1.38 ± NR C: −0.06 ± NR	A: NR B: NR^*∗*^ C: NR^*∗∗*^	Mean values of the reduction factor of log_10_ CFU demonstrated a higher reduction with chlorhexidine, followed by H_2_O_2_, which also performed better than distilled sterile water

*Studies with experimental gingivitis*								
Gusberti, 1988, Switzerland [16] Non-RCT (parallel)	Not reported Not reported Total facultative anaerobes, total cultivable microbiota, *Streptococci, actinomyces*, *A. naeslundii*, *A. viscosus*, Fusobacteria, Veillonella, and Capnocytophaga	H_2_O_2_ 1% Twice daily (15 ml for 30 s) during 21 days Smokers NR Mean age NR Baseline: NR/NR (total *n* = 10) End: NR/NR (total *n* = 10)	CHX 0.12% Twice daily (15 ml for 30s) during 21 days Smokers NR Mean age NR Baseline: NR/NR (total *n* = 11) End: NR/NR (total *n* = 11)	Flavored alcoholic placebo solution Twice daily (15 ml for 30 s) Smokers NR Mean age NR Baseline: NR/NR (total *n* = 11) End: NR/NR (total *n* = 11)	To all microbiological analyses A: NR B: NR C: NR	(60 minutes) All analyses are reported in log_10_ CFU/teeth Total facultative anaerobes A: 8.35 ± 0.08 B: 7.28 ± 0.11 C: 8.05 ± 0.17 Total cultivable microbiota A: 8.57 ± 0.09 B: 7.55 ± 0.09 C: 8.33 ± 0.15 Streptococci A: 8.14 ± 0.08 B: 7.34 ± 0.12 C: 7.73 ± 0.15 Actinomyces A: 8.14 ± 0.09 B: 5.61 ± 0.29 C: 7.72 ± 0.22 *A. naeslundii* A: 7.49 ± 0.21 B: 5.31 ± 0.29 C: 6.70 ± 0.39 *A. viscosus* A: 7.75 ± 0.22 B: 4.74 ± 0.29 C: 7.57 ± 0.24 Fusobacteria A: 5.17 ± 0.32 B: 4.87 ± 0.20 C: 6.36 ± 0.23 Veillonella A: 6.66 ± 0.18 B: 5.98 ± 0.20 C: 7.23 ± 0.23 Capnocytophaga A: 5.55 ± 0.34 B: 5.10 ± 0.18 C: 6.60 ± 0.26	A: NR B: NR^*∗*^ C: NR A: NR B: NR^*∗*^ C: NR A: NR B: NR^*∗*^ C: NR A: NR B: NR^*∗*^ C: NR A: NR B: NR^*∗*^ C: NR A: NR B: NR^*∗*^ C: NR A: NR B: NR^*∗*^ C: NR^*∗∗*^ A: NR B: NR^*∗*^ C: NR^*∗∗*^ A: NR B: NR^*∗*^ C: NR^*∗∗*^	Chlorhexidine 0.12% demonstrated a broad-spectrum activity with significant reduction in the number of both facultative and strict anaerobe However, H_2_O_2_ 1% did not affect total cultivable microbiota or facultative bacterial species such as Streptococci and Actinomyces In relation to strict anaerobes (e.g., Fusobacterium and Veillonella), H_2_O_2_ 1% was significantly less effective as compared to 0.12% chlorhexidine

Wennström, 1979 [18], Sweden Non-RCT (cross-over)	Systemically healthy Oral health Cocoid cells/straight rods, filaments, fusiforms, motile/curved rods, and spirochetes	H_2_O_2_ (1.7 g dissolved in 30 ml of tap water) Three times a day (30 ml per 2 minutes) during 14 days Smokers NR Mean age NR Baseline: NR/NR (total *n* = 14) End: NR/NR (total *n* = 14)	Not applicable	Placebo mouthwash Three times a day (30 ml per 2 minutes) during 14 days Smokers NR Mean age NR Baseline: NR/NR (total *n* = 14) End: NR/NR (total *n* = 14)	Coccoid cells +straight rods A: 88.20 ± 4.60 B: NA C: 84.20 ± 3.90 Filaments A: 4.70 ± 1.80 B: NA C: 7.00 ± 1.80 Fusiforms A: 4.00 ± 1.30 B: NA C: 4.30 ± 2.00 Motile + curved rods A: 3.00 ± 1.20 B: NA C: 4.50 ± 1.30 Spirochetes A: 0.10 ± 0.10 B: NA C: 0.10 ± 0.10	(14 days) Coccoid cells + straight rods A: 84.90 ± 4.70 B: NA C: 40.40 ± 6.00 Filaments A: 6.90 ± 2.00 B: NA C: 17.70 ± 1.80 Fusiforms A: 5.40 ± 1.80 B: NA C: 14.90 ± 1.80 Motile + curved rods A: 2.60 ± 1.80 B: NA C: 22.10 ± 4.00 Spirochetes A: 0.10 ± 0.10 B: NA C: 4.90 ± 1.80	Coccoid cells + straight rods A: 3.30 (−3.74%) B: NA C: −43.80 (−52.02%^#^)^*∗∗*^ Filaments A: 2.20 (46.81%) B: NA C: 10.70 (152.86%^#^)^*∗∗*^ Fusiforms A: 1.40 (35.00%) B: NA C: 10.60 (246.51%^#^)^*∗∗*^ Motile + curved rods A: −0.40 (−13.33%) B: NA C: 17.60 (391.11%^#^)^*∗∗*^ Spirochetes A: 0.00 (0.00%) B: NA C: 17.6 (17600%^#^)^*∗∗*^	H_2_O_2_ mouthwash prevented the colonization of filaments, fusiforms, motile, and curved rods as well as spirochetes Statistically significant differences were observed in relation to placebo

NA: not applicable; NR: not reported; RCT: randomized clinical trial; non-RCT: nonrandomized clinical trial; CHX: chlorhexidine; M: male; F: female. A: H_2_O_2_ rinse; B: chlorhexidine rinse; C: control rinse. ∆: delta of mean (final−initial mean); %: percentage of reduction. ^*∗*^Statistically significant differences between groups (H_2_O_2_ vs chlorhexidine); ^*∗∗*^statistically significant differences between groups (H_2_O_2_ vs control); ^#^statistically significant differences within group (end vs baseline score).

## Data Availability

The data supporting the current study are available from the corresponding author upon request.

## Supplementary Materials

Supplementary Materials Table S1: list of potentially relevant studies not included in the systematic review, along with the reasons for exclusion.
