# Supplementary material for: A Systematic Review of the Effect of Oral Rinsing with H2O2 on Clinical and Microbiological Parameters Related to Plaque, Gingivitis, and Microbes
Source: Int J Dent. 2020 Oct 31;2020:8841722. doi: 10.1155/2020/8841722 (PMC7648695; doi:10.1155/2020/8841722)
Supplement: Supplementary Materials — Table S1: list of potentially relevant studies not included in the systematic review, along with the reasons for exclusion. [file 8841722.f1.docx]

Table S1. List of potentially relevant studies not included in the systematic review, along with the reasons for exclusion.

N.L. Hoenderdos, N.A. Rosema, D.E. Slot, M.F. Timmerman, U. van der Velden and G.A. van der Weijden, “The influence of a hydrogen peroxide and glycerol containing mouthrinse on plaque accumulation: a 3-day non-brushing model,” *International Journal of Dental Hygiene*, vol. 7, no. 4, pp. 294-298, 2009.

|  | **Reference** | **Reason for exclusion** |
| --- | --- | --- |
| 1 | A. Binney, M. Addy and R.G. Newcombe, “The plaque removal effects of single rinsings and brushings,” *Journal of periodontology,* vol. 64, no. 3, pp. 181-185, 1993. | H_2_O_2_ was not used |
| 2 | A. Uludamar, Y.K. Özkan, T. Kadir, and I. Ceyhan, “In vivo efficacy of alkaline peroxide tablets and mouthwashes on Candida albicans in patients with denture stomatitis,” *Journal of Applied Oral Science*, vol. 18, no. 3, pp. 291-296, 2010. | H_2_O_2_ was not used |
| 3 | B.L. Dona, L.J.M.M. Gründemann, J. Steinfort, M.F. Timmerman and G.A. Van der Weijden, “The inhibitory effect of combining chlorhexidine and hydrogen peroxide on 3‐day plaque accumulation,” *Journal of Clinical Periodontology*, vol. 25, no. 11, pp. 879-883, 1998. | H_2_O_2_ was not used |
| 4 | G. Dahlen, “Effect of antimicrobial mouthrinses on salivary microflora in healthy subjects,” *European Journal of Oral Sciences*, vol. 92, no. 1, pp. 38-42, 1984. | H_2_O_2_ was not used |
| 5 | J. Dadamio, M. Van Tournout, W. Teughels, C. Dekeyser, W. Coucke and M. Quirynen, “Efficacy of different mouthrinse formulations in reducing oral malodour: a randomized clinical trial” *Journal of Clinical Periodontology*, vol. 40, no. 5, pp. 505-513, 2013. | H_2_O_2_ was not used |
| 6 | M. Fernandez y Mostajo, W.A. van der Reijden, M.J. Buijs, et al., “Effect of an oxygenating agent on oral bacteria in vitro and on dental plaque composition in healthy young adults,” *Frontiers in Cellular and Infection Microbiology*, vol. 4, no. 95, 2014. | H_2_O_2_ was not used |
| 7 | J. Moran, M. Addy, W. Wade, S. Milson, R. McAndrew, and R.G. Newcombe, “The effect of oxidising mouthrinses compared with chlorhexidine on salivary bacterial counts and plaque regrowth,” *Journal of Clinical Periodontology*, vol. 22, no. 10, pp. 750-755, 1995. | H_2_O_2_ was not used |
| 8 | M.K. Chadha, J.R. Taneja and B.R. Vacher, “Effect of an antiseptic mouthwash on plaque accumulation,” *Journal of periodontology*, vol. 49, no.5, pp. 266-268, 1978. | H_2_O_2_ was not used |
| 9 | A. Ramesh, J.T. Thomas, N.P. Muralidharan, and S.S. Varghese, “Efficacy of adjunctive usage of hydrogen peroxide with chlorhexidine as preprocedural mouthrinse on dental aerosol,” *National Journal of Physiology, Pharmacy and Pharmacology*, vol. 5, no. 5, pp. 431-435, 2015. | H_2_O_2_ and other substance in the same group |
| 10 | A.Z. Siddiqui, S. Vellappally, H. Fouad, and S.M. Muckarrum, “Bactericidal and clinical efficacy of photochemotherapy in acute necrotizing ulcerative gingivitis,” *Photodiagnosis and Photodynamic Therapy*, vol. 29, pp. 101668, 2020. | H_2_O_2_ and other substance in the same group |
| 11 | C. Occhipinti, “Effect of a Hydrogen-Peroxide and Hyaluronic-Acid Mouthwash (BMG0703) in the Treatment of Periodontitis”, *Clinical Trials* protocol. ClinicalTrials.gov Identifier: NCT04446533, 2020. | H_2_O_2_ and other substance in the same group |
| 12 | Comparison clinical outcomes and patient satisfaction between chlorhexidine mouthrinses and chlorhexidine mixed with hydrogen peroxide mouthrinses, Randomized clinical trial protocol, ID Number: CN-01993300, 2019 | H_2_O_2_ and other substance in the same group |
| 13 | D. Steinberg, I. Heling, I. Daniel, and I. Ginsburg, “Antibacterial synergistic effect of chlorhexidine and hydrogen peroxide against Streptococcus sobrinus, Streptococcus faecalis and Staphylococcus aureus” *Journal of Oral Rehabilitation*, vol. 26, no. 2, pp. 151-156, 1999. | H_2_O_2_ and other substance in the same group |
| 14 | P. Jhingta, A. Bhardwaj, D. Sharma, N. Kumar, V.K. Bhardwaj and S. Vaid, “Effect of hydrogen peroxide mouthwash as an adjunct to chlorhexidine on stains and plaque,”. *Journal of Indian Society of Periodontology*, vol. 17, no. 4, pp. 449, 2013. | H_2_O_2_ and other substance in the same group |
| 15 | P.D. Jaña, L.I. Yévenes, and A.S. Rivera, “Estudio Clínico Comparativo entre Colutorio de p-clorofenol y peróxido de hidrógeno con Colutorio de Clorhexidina al 0.12% en el Crecimiento de Placa Microbiana y Gingivitis,” *Revista Clínica de Periodoncia, Implantología y Rehabilitación Oral*, vol. 3, no. 2, pp. 65-68, 2010. | H_2_O_2_ and other substance in the same group |
| 16 | L.W. Slanetz and E.A. Brown, “Studies of the Effect of Glycerite of Hydrogen Peroxide Upon the Numbers of Oral Microorganisms',” *Journal of Dental Research*, vol. 25, no. 4, pp. 223-230, 1946. | Absence of control group |
| 17 | L.W. Slanetz and E.A. Brown, “Studies on the numbers of bacteria in the mouth and their reduction by the use of oral antiseptics,” *Journal of Dental Research*, vol. 28, no. 3, pp. 313-323, 1949. | Absence of control group |
| 18 | F.G. Lima, T.A. Rotta, S. Penso, S.S. Meireles and F.F. Demarco, “In vitro evaluation of the whitening effect of mouth rinses containing hydrogen peroxide,” *Brazilian Oral Research*, vol. 26, no. 3, pp. 269-274, 2012. | *In vitro* study |
| 19 | M.F. y Mostajo, R.A. Exterkate, M.J. Buijs, W. Crielaard and E. Zaura, “Effect of mouthwashes on the composition and metabolic activity of oral biofilms grown in vitro,” *Clinical Oral Investigations*, vol. 21, no. 4, pp. 1221-1230, 2017. | *In vitro* study |
| 20 | S. Hasheminia, A.R. Farhad, M. Saatchi, and M. Rajabzadeh, “Synergistic antibacterial activity of chlorhexidine and hydrogen peroxide against Enterococcus faecalis,” *Journal of Oral Science*, vol. 55, no. 4, pp. 275-280, 2013. | *In vitro* study |
| 21 | S.Y. Lee, “Effects of chlorhexidine digluconate and hydrogen peroxide on Porphyromonas gingivalis hemin binding and coaggregation with oral streptococci,” *Journal of Oral Science*, vol. 43, no.1, pp. 1-7, 2001. | *In vitro* study |
| 22 | Evaluation of a dentifrice with natural ingredients in the prevention of plaque and gingivitis, Randomized clinical trial protocol. Accession Number: ICTRP NTR1215, 2008 | Mouthwash was not used |
| 23 | M.K. Yamalik, S. Yücetas, and U. Abbasoglu, “Effects of various antiseptics on bacteremia following tooth extraction,” *The Journal of Nihon University School of Dentistry*, vol. 34, no. 1, pp. 28-33, 1992. | Mouthwash was not used |
| 24 | I. Pai, S. Lo, S. Brown and A.G. Toma, A. G., “Does hydrogen peroxide mouthwash improve the outcome of secondary post-tonsillectomy bleed? A 10-year review,” *Otolaryngology—Head and Neck Surgery*, vol. 133, no. 2, pp. 202-205, 2005. | Outcomes were not assessed |
| 25 | K. Hutchins, G. Karras, J. Erwin and K.L. Sullivan, “Ventilator-associated pneumonia and oral care: a successful quality improvement project. *American Journal of Infection Control*, vol. 37, no. 7, pp. 590-597, 2009. | Outcomes were not assessed |
| 26 | M. Addy, F. Al‐Arrayed and J. Moran, “The use of an oxidising mouthwash to reduce staining associated with chlorhexidine: studies in vitro and in vivo,” *Journal of Clinical Periodontology*, vol. 18, no. 4, pp. 267-271, 1991 | Outcomes were not assessed |
| 27 | M. Nobahar, M.R. Razavi, F. Malek and R. Ghorbani, “Effects of hydrogen peroxide mouthwash on preventing ventilator-associated pneumonia in patients admitted to the intensive care unit,” *Brazilian Journal of Infectious Diseases*, vol. 20, no. 5, pp. 444-450, 2016. | Outcomes were not assessed |
| 28 | M. Gosau, S. Hahnel, F. Schwarz, T. Gerlach, T.E. Reichert and R. Bürgers, “Effect of six different peri‐implantitis disinfection methods on in vivo human oral biofilm,” *Clinical Oral Implants Research*, vol. 21, no.8, pp. 866-872, 2010. | Study with dental implants |
| 29 | A. Chandu, C. Stulner, A.M. Bridgeman and A.C. Smith, “Maintenance of mouth hygiene in patients with Oral cancer in the immediate post‐operative period,” *Australian Dental Journal*, vol. 47, no. 2, pp. 170-173, 2002. | Review |
| 30 | N. Hossainian, D.E. Slot, F. Afennich and G.A. Van der Weijden, G. A., “The effects of hydrogen peroxide mouthwashes on the prevention of plaque and gingival inflammation: a systematic review,” *International Journal of Dental Hygiene*, vol. 9, no. 3, pp. 171-181, 2011. | Review |
| 31 | C. Hasenau, B.P.E. Clasen and D. Roettger, “Anwendung einer standardisierten Mundpflege zur Prophylaxe und Therapie einer Mukositis hei Patienten während der Radiochemotherapie von Kopf-Hals-Malignomen” *Laryngologie, Rhinologie, Otologie und ihre Grenzgebiete*, vol. 67, no. 11, pp. 576-579, 1988. | Not found |
| 32 | C.M. Jones, A.S. Blinkhorn and E. White, “Hydrogen peroxide, the effect on plaque and gingivitis when used in an oral irrigator,” *Clinical Preventive Dentistry*, vol. 12, no. 5, pp. 15-18, 1990. | Not found |
| 33 | C.W. Chapek, O.K. Reed, and P.A. Ratcliff, “Management of periodontitis with oral-care products,” *Compendium (Newtown, Pa.)*, vol. 15, no. 6, pp. 740-742, 1994. | Not found |
| 34 | F.P. Clausen, “Local treatment of acute necrotizing gingivitis with Ascoxal (R): clinical experiences from treatment of military personnel,” *Tandlaegebladet*, vol. 70, no. 12, pp. 1009, 1966. | Not found |
| 35 | G. Ricciardi, G. Quaranta, N. Milani and P. Laurenti, “Antibacterial effective of an oral rinse containing hydrogen peroxide and hyaluronic acid,” *Dental Cadmos*, vol. 78, no. 10, pp. 53-56, 2010. | Not found |
| 36 | H. Chen, “Reducing bacterial aerosols by ultrasonic scaling with Koutai or 1.5% H2O2 pre-rinse” *Zhonghua kou qiang yi xue za zhi= Zhonghua kouqiang yixue zazhi= Chinese journal of stomatology*, vol. 35, no. 5, pp. 381-382, 2000. | Not found |
| 37 | M.B. Tombes and B. Gallucci, “The effects of hydrogen peroxide rinses on the normal oral mucosa” *Nursing* *Research*, vol. 42, no. 6, pp. 332-337, 1993. | Not found |
| 38 | O. Shibly, S.G. Ciancio, M. Kazmierczak, et al., “Clinical evaluation of the effect of a hydrogen peroxide mouth rinse, sodium bicarbonate dentifrice, and mouth moisturizer on oral health,” *The Journal of Clinical Dentistry,* vol. 8, no. 5, pp. 145-149, 1997. | Not found |
| 39 | P. Gängler and W. Staab, “2-year clinically controlled study of plaque prevention with chlorhexidine digluconate and hydrogen peroxide in marginal periodontitis” *Zahn-, Mund-, und Kieferheilkunde mit Zentralblatt*, vol. 73, no. 3, pp. 253, 1985. | Not found |
| 40 | R.A. Winer, H.H. Chauncey, and R.I. Garcia, “Effect of Peroxyl mouthrinse on chlorhexidine staining of teeth,” *The Journal of Clinical Dentistry*, vol. 3, no. 1, pp. 15-18, 1991. | Not found |
| 41 | S.K. Rich, A.K. Horikoshi, and M.G. Newman, “Longitudinal effects of an oxygenating agent on clinical indices and oral microbiota,” *Clinical Preventive Dentistry*, vol. 2, no. 1, pp. 13-17, 1980. | Not found |
| 42 | J. Yaghini, “Evaluation of different treatment regimes of hydrogen proxide and chlorhexidine mouthrinses on dental stain and plaque,” Randomized clinical trial protocol, Accession Number: ICTRP IRCT20101006004877N28, 2020. | Study protocol (data not provided or not available) |
| 43 | S.A. Gansky, “Antiseptic Mouthwash / Pre-Procedural Rinse on SARS-CoV-2 Load (COVID-19) (AMPoL),” *Clinical Trials Protocol*, ClinicalTrials.gov Identifier: NCT04409873, 2020 | Study protocol (data not provided or not available) |
